# Measurement of liver iron by magnetic resonance imaging in the UK Biobank population

**DOI:** 10.1371/journal.pone.0209340

**Published:** 2018-12-21

**Authors:** Andy McKay, Henry R. Wilman, Andrea Dennis, Matt Kelly, Michael L. Gyngell, Stefan Neubauer, Jimmy D. Bell, Rajarshi Banerjee, E. Louise Thomas

**Affiliations:** 1 Perspectum Diagnostics, Oxford, United Kingdom; 2 Research Centre for Optimal Health, School of Life Sciences, University of Westminster, London, United Kingdom; 3 Oxford Centre for Clinical Magnetic Resonance Research (OCMR), Division of Cardiovascular Medicine, Radcliffe Department of Medicine, University of Oxford, Oxford, United Kingdom; McMaster University, CANADA

## Abstract

The burden of liver disease continues to increase in the UK, with liver cirrhosis reported to be the third most common cause of premature death. Iron overload, a condition that impacts liver health, was traditionally associated with genetic disorders such as hereditary haemochromatosis, however, it is now increasingly associated with obesity, type-2 diabetes and non-alcoholic fatty liver disease. The aim of this study was to assess the prevalence of elevated levels of liver iron within the UK Biobank imaging study in a cohort of 9108 individuals. Magnetic resonance imaging (MRI) was undertaken at the UK Biobank imaging centre, acquiring a multi-echo spoiled gradient-echo single-breath-hold MRI sequence from the liver. All images were analysed for liver iron and fat (expressed as proton density fat fraction or PDFF) content using LiverMultiScan. Liver iron was measured in 97.3% of the cohort. The mean liver iron content was 1.32 ± 0.32 mg/g while the median was 1.25 mg/g (min: 0.85 max: 6.44 mg/g). Overall 4.82% of the population were defined as having elevated liver iron, above commonly accepted 1.8 mg/g threshold based on biochemical iron measurements in liver specimens obtained by biopsy. Further analysis using univariate models showed elevated liver iron to be related to male sex (p<10^−16^, r^2^ = 0.008), increasing age (p<10^−16^, r^2^ = 0.013), and red meat intake (p<10^−16^, r^2^ = 0.008). Elevated liver fat (>5.6% PDFF) was associated with a slight increase in prevalence of elevated liver iron (4.4% vs 6.3%, p = 0.0007). This study shows that population studies including measurement of liver iron concentration are feasible, which may in future be used to better inform patient stratification and treatment.

## Introduction

The burden of chronic liver disease and its impact on morbidity in terms of liver failure, fibrosis and end-stage cirrhosis and, ultimately, mortality is increasing, reportedly at much higher rates in the UK than other western European countries [1,2]. Whilst the aetiology of liver disease varies, common causes include viruses (e.g. Hepatitis C), autoimmune conditions, as well as lifestyle related factors including obesity and alcohol. The consequence of an increasingly obese population has a significant impact both on the prevalence and severity of non-alcoholic fatty liver disease (NAFLD) and non-alcoholic steatohepatitis (NASH), as well as the progression of both Hepatitis C and alcohol related liver diseases [3, 4].

Iron overload is traditionally associated with genetic disorders such as hereditary haemochromatosis (HH) or as a result of repeated blood transfusions. However elevated levels of liver iron are now often associated with metabolic diseases including insulin resistance, type-2 diabetes and NAFLD [5–7]. This has been referred to as insulin resistance-associated hepatic iron overload [8,9] or as it is now more commonly known dysmetabolic iron overload syndrome (DIOS). Unlike HH where liver iron concentrations are severely raised, with DIOS mild increases in iron stores are more common [6]. It has been suggested that one third of patients with NAFLD have DIOS [6], with others reporting even greater prevalence [7].

Irrespective of its causes, identifying individuals storing excess liver iron and assessing its prevalence within the population is paramount since elevated liver iron is associated with the development of fibrosis and cirrhosis, and thought to be involved in the progression from fibrosis to hepatocellular carcinoma [10]. There is however ongoing debate as to whether this progression is driven by the mild to moderate levels of liver iron typically observed in subjects with NAFLD or the high levels of serum ferritin that are often observed [11]. To fully understand the health implications of elevated levels of liver iron content, an assessment of its prevalence within the population is necessary as well as developing population appropriate methods for its measurement which will allow early identification and monitoring of response to interventions, prior to the onset of organ failure.

Liver biopsy is the gold standard for assessment of liver iron, with a normal levels of liver iron reported as <1.8 mg/g dry weight [12,13]. However, liver biopsy is not clinically recommended except in cases where advanced fibrosis is suspected [14,15]. The need to regularly measure liver iron content commonly relies on indirect blood-based biomarkers such as serum ferritin. However, ferritin levels can be influenced by liver damage and inflammation, and in many cases do not correlate well with liver iron stores [16]. This has necessitated the development of robust non-invasive techniques for the measurement of liver iron based on magnetic resonance imaging (MRI) [17,18].

MRI measurements of liver iron are based on the principle that the rate of MR signal decay is influenced by the levels of iron in the tissue; more iron present results in a faster signal decay. Multiple calibration studies have been published, comparing measurements of liver iron by MRI and tissue biopsy, establishing MRI as a suitable method for the assessment of liver iron in both healthy and iron-overload subjects [17,19–24]. Despite numerous studies utilising MRI to measure liver iron, these have generally been conducted in relatively small and well defined patient populations. Few studies have characterised the distribution of liver iron within the general population. In the present study, we describe the measurement of liver iron in a population >9,000 individuals within the UK Biobank Cohort, and assess its distribution in relation to age, sex, body habitus and liver fat content.

## Patients and methods

### Study design

The UK Biobank (UKB) imaging study is a large prospective study of people aged between 40–69 years (at the time of initial recruitment in 2006–2010), with a planned 100,000 subjects to be recruited from the wider 500,000-strong general UK Biobank Cohort. This study assesses the first 9108 subjects from the UKB imaging enhancement protocol, acquired between 2014 and 2016, with patient meta-data obtained through UK Biobank Access Application number 9914. The UK Biobank has approval from the North West Multi-Centre Research Ethics Committee (MREC), and obtained written informed consent from all participants prior to the study.

We performed a cross-sectional study in UK Biobank (52.4% female, mean age 61.4 [44–73] years, 96.7% white), to determine how liver iron varies according to sex, age, body mass index (BMI) and liver fat.

### Imaging protocol

Participants were scanned at the UK Biobank imaging centre in Cheadle (UK) using a Siemens 1.5T Magnetom Aera. A multi-echo spoiled gradient-echo single-breath-hold MRI sequence was acquired as a single transverse slice captured through the centre of the liver superior to the porta hepatis. This sequence is part of the LiverMultiScan protocol from Perspectum Diagnostics (UK) which forms part of the UK Biobank abdominal imaging protocol [25,26].

### Image analysis

The data was analysed using the LiverMultiScan Discover software by a team of trained analysts, blinded to any subject variables. Analysts selected three 15mm diameter circular regions of interest (ROIs), to cover a representative sample of the liver parenchyma, avoiding vessels, bile ducts and other organs. Mean T2*, liver iron, and PDFF were calculated. The repeatability and reproducibility of the image analysis is described in the S1 Appendix.

### Presentation of liver iron

There is little consensus within the literature regarding the best protocol for the measurement of liver iron by MRI. Liver iron measures obtained by MRI are sometimes reported as T2* and/or R2* or converted to iron in mg/g, utilising a variety of formulas (S1 Table) based on regression analysis in studies where both MRI and biopsy data were available [17,19–23,27]. The impact on the final results arising from the use of these formulas can be seen in S2 Table. In the present study values are presented as mg/g as described by Wood et al [19], with a cut-off of <1.8 mg/g dry weight for healthy liver iron [12,13], which equates to a R2* cut-off of 62.7 sec^-1^. Wherever appropriate, T2* and R2* values are also shown (S2 Table).

### Statistical methodology

Summary data are presented as means, medians and quartiles. Assessment of difference of prevalence of elevated liver iron levels by stratified groups was completed using chi-squared tests. Subjects were stratified into age (40–49, 50–59, 60–69 and 70–79 years) and BMI groups (<20 kg/m^2^, 20–24.9 kg/m^2^ (normal), 25–29.9 kg/m^2^ (overweight), 30–34.9 kg/m^2^ (obese), and >35 kg/m^2^ (very obese). Repeatability and reproducibility were assessed with intra-class correlation (ICC) [28] and Bland-Altman analyses [29] (presented in S1 Appendix). Linear models were fitted using R. Liver fat values were log transformed for the purposes of the models, since this transformation resulted in an improved fit, with more normal residuals. Categorical variables were modelled as fixed effects. Iron values were capped at 3mg/g in plots but left untransformed for all other analysis. For beef intake, the results were recoded to give groups with sufficient numbers. The "2–4 times a week", "5–6 times a week", and "Once or more daily" labels were combined into a single level of “More than once a week”. The "Do not know" and "Prefer not to answer" levels were set to missing values. The remaining three levels, “Never”, "Less than once a week", and "Once a week", were left unchanged. Statistical analyses were undertaken using Python 3.6 (Python Software) and R 3.4.4 (R Core Team) [30, 31].

## Results

Liver iron was measured in 8865 (97.33%) of the 9108 MRI datasets available. Of the 243 (2.67%) cases not deemed of sufficient quality for analysis; 170 had artefacts, in 41 cases the slice was incorrectly positioned and did not include usable liver, 15 could not be linked to their metafiles and 10 were rejected during quality control processing due to missing or corrupted files. A further 7 were rejected as their T2* values were below the limits of detection.

The demographics of the population included in the final analysis are presented in Table 1.

**Table 1:** General population statistics.

	Subjects (n)	Subjects (%)
Total population	8865	
**Sex**		
Male	4219	47.6
Female	4646	52.4
**Age** (years)		
40–49 years	551	6.2
50–59 years	2651	29.9
60–69 years	4161	46.9
70–79 years	1093	12.3
Age not available	409	4.6
**BMI** (kg/m^2^)		
<20 kg/m^2^	294	3.3
20–24.9 kg/m^2^	3189	36.0
25–29.9 kg/m^2^	3725	42.0
30–34.9 kg/m^2^	1222	13.8
>35 kg/m^2^	405	4.63
BMI not available	30	0.3
**Ethnicity**		
White	8512	96.0
Mixed	47	0.5
Asian	107	1.2
Black	51	0.6
Chinese	29	0.3
Other ethnicity	37	0.4
Ethnicity not available or subject preferred not to answer	82	0.9

**Fig 1:**
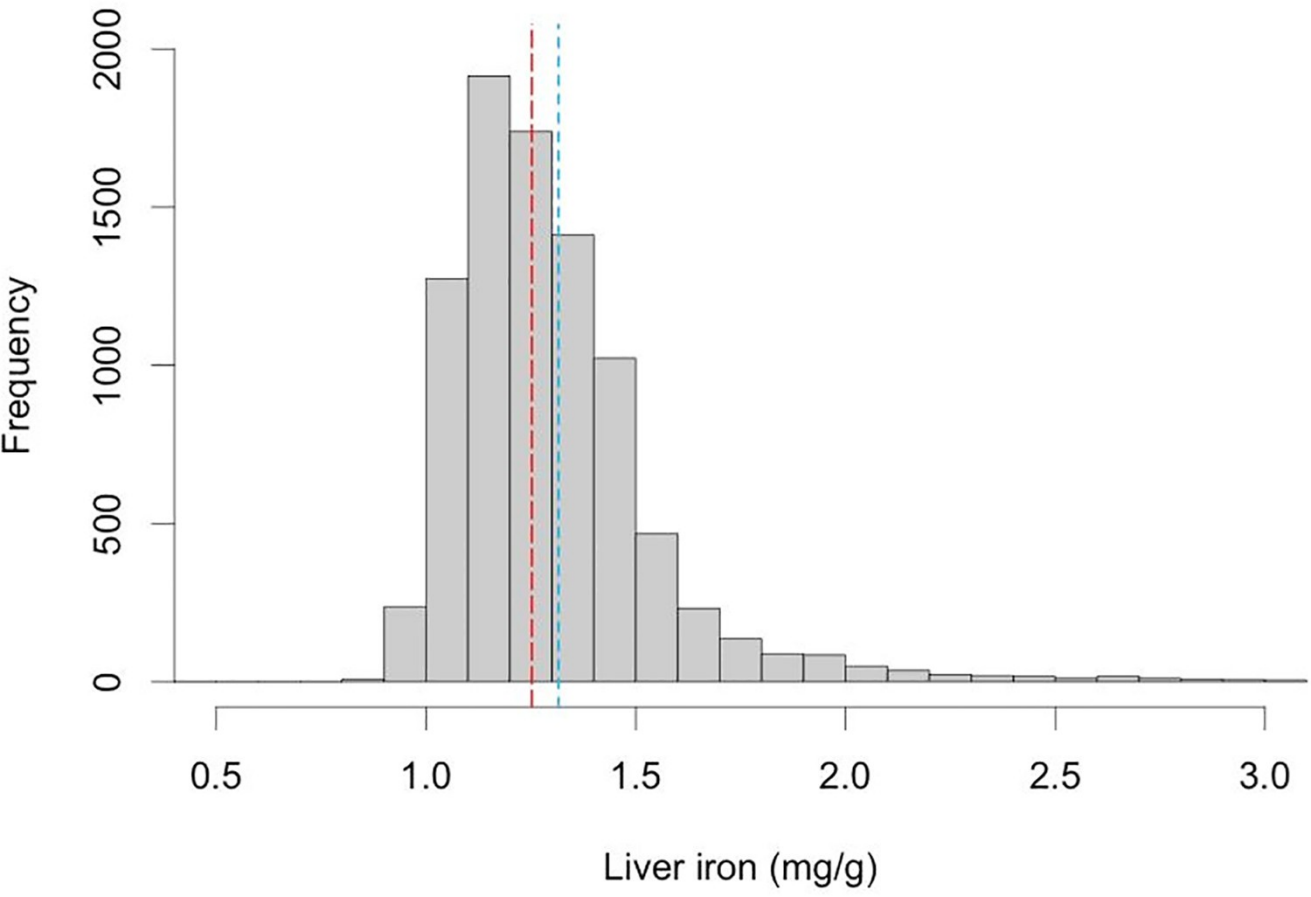
Distribution of liver iron concentration (liver iron) within the UK Biobank population. Median value (1.25 mg/g) shown in red, mean (1.32 mg/g) in blue. There were 52 individuals with liver iron > 3mg/g, who are not shown in this plot.

The mean and median liver iron for this cohort was 1.32 ± 0.32 mg/g and 1.25 mg/g (min: 0.85 max: 6.44 mg/g) respectively (Table 2). The distribution of liver iron within the population is presented in Fig 1 (and also shown as a T2* distribution in S1 Fig). Overall 4.82% of the population were defined as having elevated liver iron, above the commonly accepted 1.8mg/g threshold [12, 13].

**Table 2:** Summary statistics for liver iron.

	liver iron mg/g (Wood [19])
Mean	1.32
St. dev	0.32
5^th^ Percentile	1.02
25^th^ Percentile	1.14
Median	1.25
75^th^ Percentile	1.40
95^th^ percentile	1.78

All data expressed as mg/g dry weight

For a ‘reference’ population, defined as those individuals with BMI <25 kg/m^2^, and PDFF <5%, the 95% range was 0.98–2.06 mg/g dry weight (this corresponds to a T2* range of 32.2–13.6 ms, and a R2* range of 31.02–74.87 s^-1^). This population had a very similar average age (61.37 years) to the whole cohort (61.10 years).

### Relationship with proton density fat fraction (PDFF)

**Fig 2:**
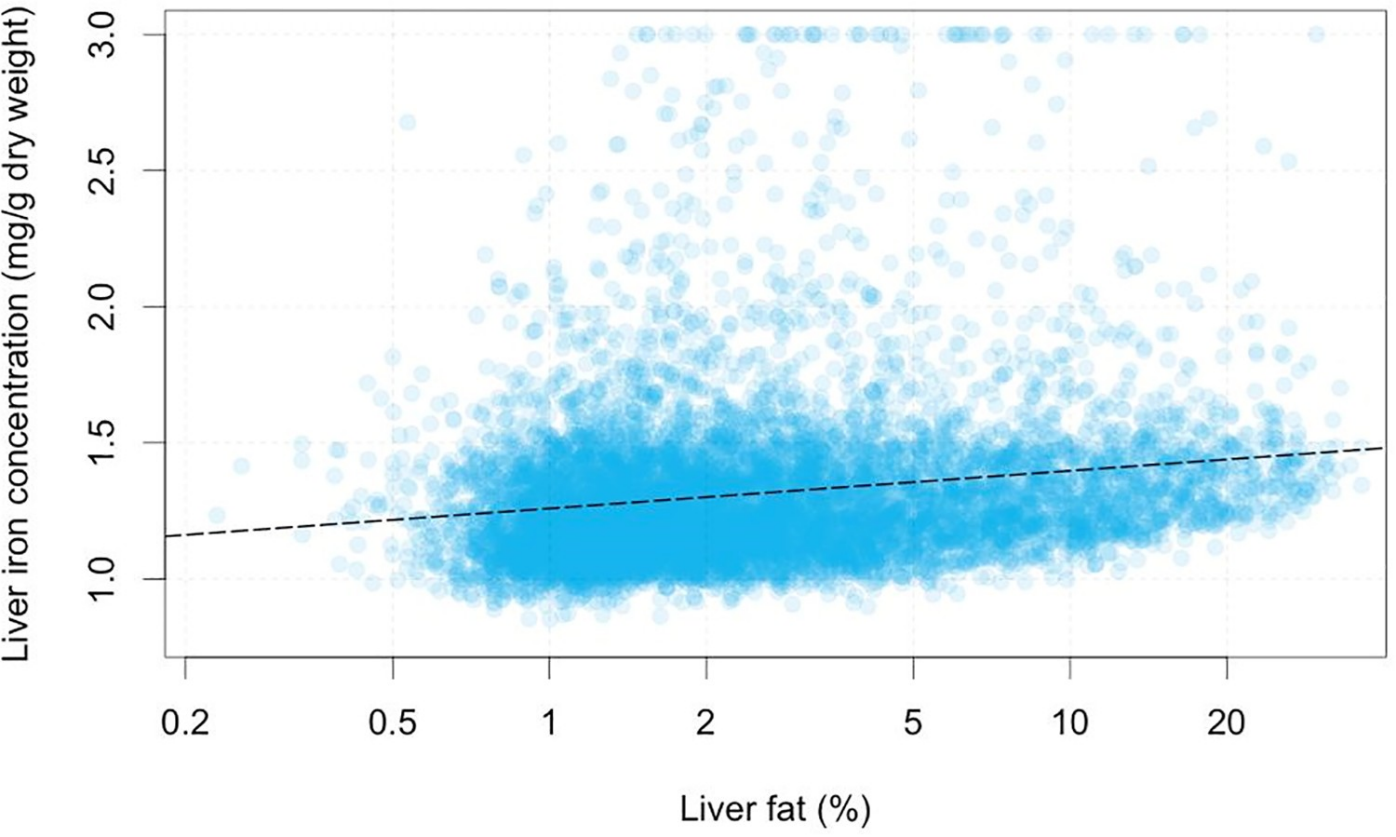
Correlation of liver iron concentration and liver fat (MRI proton density fat fraction) in the UK Biobank population.

Subjects were further stratified according to their liver fat content (PDFF), derived from MRI. Overall 20.6% (1826) of participants had elevated PDFF (>5.6% [32]), There was a weak but significant relationship between liver iron and log(PDFF) (r^2^ = 0.0496, p<10^−16^, Spearman’s. r^2^ = 0.0279 Pearson’s, p<10^−16^), as shown in Fig 2. The estimated regression coefficient was 0.0595 mg/g/log(%), indicating that a doubling of liver fat corresponds, on average to an increase of 0.0412 mg/g liver iron. This means that an individual with a PDFF of 1% and another with a PDFF of 20% (roughly the extremes of the data) would have an average difference in liver iron content of 0.18mg/g.

### Age and sex

**Fig 3:**
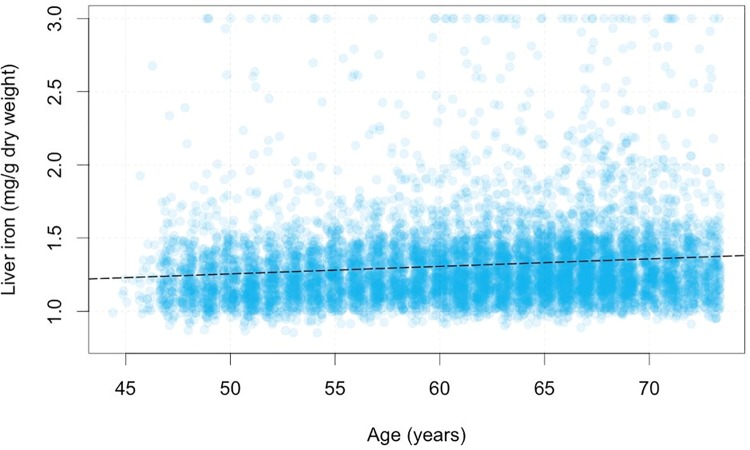
Correlation of liver iron concentration (mg/g dry weight) with age. Iron values above 3mg/g have been capped to 3mg/g in the plot, but not as part of the linear fit. Dashed black line shows line of best fit in univariate linear model.

Age was positively correlated with liver iron (p<10^−16^, r^2^ = 0.013), increasing 0.00512mg/g/year (Fig 3). Males had, on average, 0.0586 mg/g more liver iron than females (p < 10^−16^, r^2^ = 0.008). Prevalence of elevated liver iron content was significantly more common in male compared with female subjects (6.38% vs 3.4%, p<0.0001).

### Relationship with body mass index

**Fig 4:**
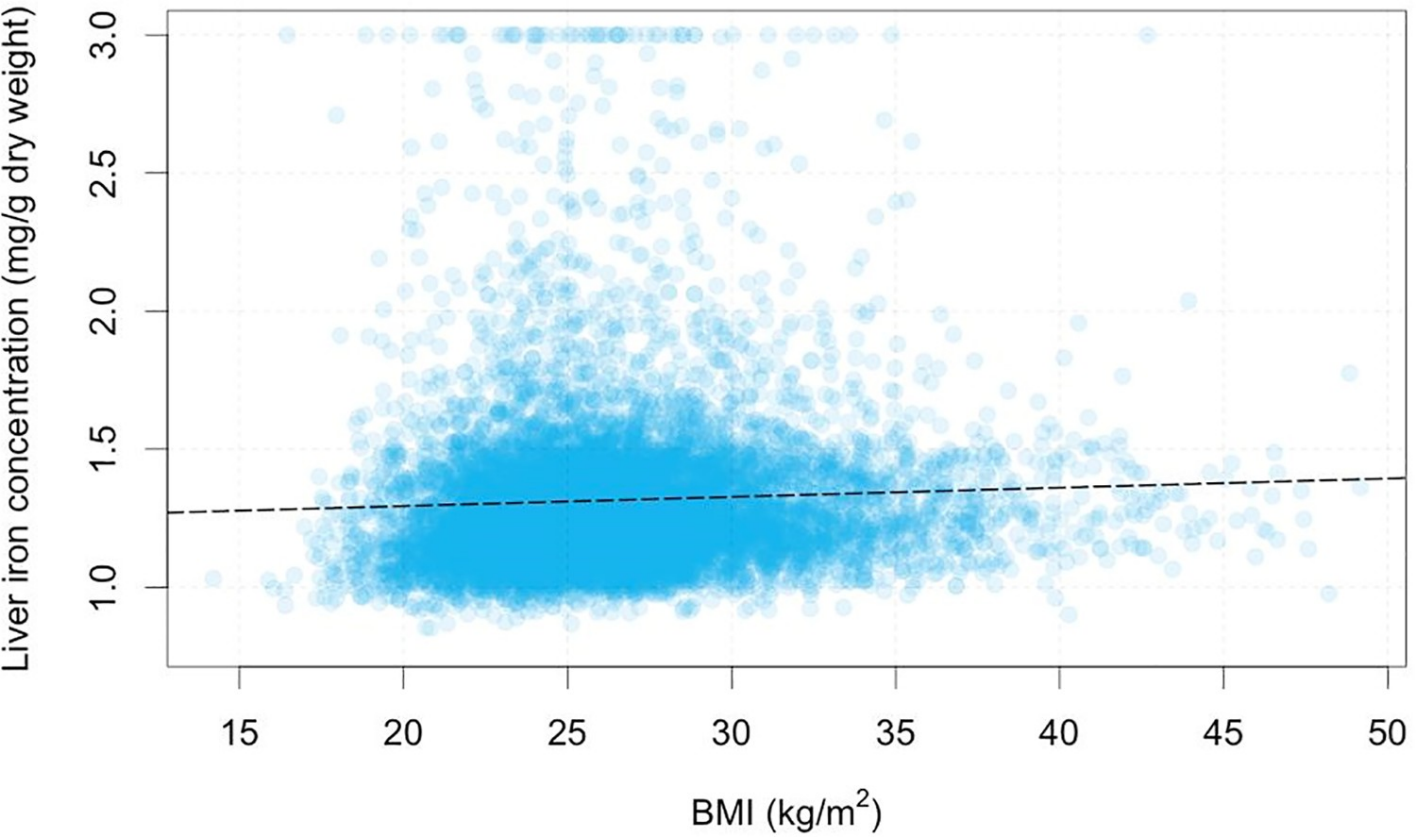
Graph showing distribution of BMI (kg/m^2^) in the UK Biobank population against liver iron (mg/g). Iron values above 3mg/g have been capped to 3mg/g in the plot, but not as part of the linear fit. Dashed black line shows line of best fit in univariate linear model.

The impact of BMI on liver iron was also investigated; in this cohort 39.3% of the population had a normal BMI, 42.0% were overweight and 18.4% were categorised as obese. There was a small yet significant correlation between BMI and liver iron (r = 0.05, P<0.0001). The variation of liver iron against BMI is shown in Fig 4.

### Ethnicity

While the cohort was predominantly Caucasian, (n = 8512) there were some Asian (n = 107), Black (n = 51) and mixed-race individuals represented to enable preliminary analysis. This showed a statistically significant difference (p<0.05) in liver iron between Caucasian (median 1.32 mg/g, range 1.14–1.41 mg/g) and Asian (median 1.26 mg/g, range 1.09–1.33 mg/g) subjects, with no significant differences between the other ethnic groups.

### Diet

There were significant differences in liver iron in relation to self-reported frequency of beef intake. A fixed-effects model relating liver iron to this variable (recoded into four groups), with the “Never” group as the baseline. The three other groups, “less than once a week”, “once a week”, and “more than once a week”, had significantly more iron (p < 10^−16^ in all cases), with 0.080, 0.108 and 0.122 mg/g higher liver iron respectively.

### Multivariate analysis

Age, sex, BMI, liver fat, and beef intake were used to create a combined model (S3 Table). Interaction terms were included in the initial model fit, and then backward selection was used to build the final model. In the final model, no interaction term was included, but each of age, sex, BMI, log(PDFF), and beef intake were included. These were all found to be significantly correlated (p < 10^−7^ in all cases). The coefficients from the model are shown in S3 Table. The r^2^ for the model was 0.053. In the multivariate model, the coefficient for log(PDFF) was larger than the univariate fit, but all other variables, except BMI, had lower coefficients in the multivariate model than in their respective univariate models. The BMI coefficient was changed in sign with respect to the univariate model.

## Discussion

The findings from this cross-sectional study into liver iron concentration in the UK Biobank revealed that 4.82% of the subjects have elevated liver iron (>1.8 mg/g) and that age, sex, ethnicity, dietary intake of beef, BMI, and liver fat, had a significant impact.

The use of MRI based methodology to assess liver iron has enabled measurement in populations where biopsy may not have been appropriate. Whilst previous studies of liver iron have generally focussed on small well-defined patient cohorts, there has recently been a similar population based study of liver iron in a German cohort (n = 2561) [33]. That study reported a prevalence of elevated levels of liver iron of 17.4%, considerably higher than that reported in the present study (<5%). The reason(s) underlying this difference are unclear but may relate to the nature of the cohort under study. For example, prevalence of fatty liver disease within the UK Biobank population was lower (20%) [26], than that reported by Kuhn et al (42.2%) [33]. Given that in the present study we observed a higher proportion of elevated liver iron levels in subjects with high PDFF, a far greater prevalence of elevated liver iron would also be expected within the German cohort. However methodological differences in MR acquisition and analysis, as well as the thresholds used for defining elevated levels of liver iron cannot be discounted, as they could potentially contribute significantly to these differences. Kuhn’s study reports a considerably lower median R2* (34.4s^-1^) and hence lower median liver iron, than measured in our study (41.4 s^-1^). Moreover, reanalysis of our dataset using their stated cut-off for elevated levels of liver iron, defined as R2*>41 sec^-1^ [34], would suggest that 51.5% of the subjects in the UK Biobank would be deemed as having elevated levels of liver iron. Interestingly, others have suggested a quite different cut-off for the normal R2* threshold, (see S4 Table for a summary of this data). Applying 57 s^-1^ cut-off suggested for multi-echo Dixon sequences gives an UKBB prevalence of elevated liver iron levels of 7.5% [17]. Using 65 s^-1^ as suggested by Paisant et al [27] would give a prevalence of elevated levels of liver iron within the UK Biobank of 4.2%.

Moreover, using the <70 s^-1^ suggested by Henninger et al [20] would result in a prevalence of elevated levels of liver iron of 3.0%. However, sequence dependence for cut-offs are known, with 3D multi-echo Dixon sequences showing lower R2* values than 2D ME GRE sequences [17]. Clearly choice of threshold to define elevated levels of liver iron will have considerable impact on reported prevalence in population studies. Whilst these published thresholds have been determined from extensive calibration studies combining MRI and biopsy data, many are still dependent on several factors including choice of sequence, field strength and cohort. Our overall finding of a prevalence of elevated levels of liver iron of just under 5%, based on biopsy related cut-off, are in line with previously published studies of explant pathology reports which found mildly elevated levels of liver iron in 5.6% of patients studied [35].

Correlations were observed between liver iron and several variables tested here. The effect sizes were consistent between the univariate models and the combined model, except for the BMI effect. The r^2^ was 0.05, indicating that these variables only explain a small amount of the population variability, so it is likely that there are more drivers of liver iron concentration than those discussed here. It is possible that the factors we have investigated in this model may be surrogates for factors that affecting iron absorption or liver iron deposition and not causative factors, further longitudinal and/or interventional studies will be required to understand this further. The change in sign of the BMI effect between the univariate and combined models, is likely a result of the linear regression using correlated variables (BMI correlates with log(PDFF), r^2^ = 0.31), and the resulting impact of this on the effect estimates of the linear model.

In the present study we report that liver iron increased significantly with age; 0.005 mg/g/year in the univariate model, and 0.004 mg/g/year in the multivariate model. We did not observe a different age-related trend in the male and female participants. An age-related increase in liver iron has previously been reported [33], the hepatic iron index is based on the concept that liver iron increases with age in HH [13]. Some of these have suggested that liver iron increases until the age of 50 years and then declines [34, 36]. However there have also been conflicting studies that have reported no age association between liver iron and HH [37, 38]. Further work may be necessary to fully understand this association, however there are relatively few individuals with diagnosed HH within the UKBiobank study; 123 in the overall cohort of 500,000, equating to approximately two individuals within the imaging cohort included in the present study. The very small numbers of HH individuals appears to support the view that the aetiology of the elevated levels of liver iron observed within the UKBB cohort is more likely associated with DIOS, undiagnosed HH, or other factors such as thalassemia. Currently, it is unclear whether this relatively small increase in liver iron can be regarded as clinically relevant. However, several studies have suggested a link between liver iron and development of fibrosis in subjects with NAFLD, although the underlying mechanisms may depend on whether the liver iron accumulation was hepatocellular or parenchymal [39, 40]. Consequently, it may be that in subjects with NAFLD even relatively small increases in liver iron may be significant and warrant further investigation.

Liver iron was significantly higher in male compared with female subjects in the UK Biobank cohort, although in absolute terms this difference was small. Prevalence of elevated liver iron was also significantly more common in male subjects compared with female subjects. This confirms the findings of previous studies which have reported similar sex differences in liver iron levels and prevalence of elevated levels of liver iron [33, 40]. This is further supported by studies showing more frequent prevalence of DIOS in males [8]. It has been shown that iron levels in females are general low until the menopause when they increase considerably [41], one suggestion is the gradual increases in body iron storage that occurs throughout aging in men are ameliorated by the effects of menstruation [42], although other studies suggest that this change may be accounted for by changes in dietary habits [43].

Ethnicity appeared to have a small yet significant impact of liver iron levels, which were significantly elevated in Caucasian compared with Asian subjects. Given the differences in the median values are very small, this may not be clinically significant, however reports of hemochromatosis within Asian populations are low [44], therefore the impact of ethnicity of liver iron levels may warrant further investigation.

There was a very weak relationship between liver iron and BMI, with prevalence of elevated liver iron levels being less common in subjects with a BMI less than 20 kg/m^2^. Relatively few studies have explored the relationship between BMI and elevated liver iron levels and available data are conflicting. Zheng et al reported a positive association between liver iron and BMI [45]; however, this is at odds with the findings of Nelson et al who reported lower BMI to be associated with increased liver iron [40]. These differences may be accounted for by the fact that our cohort was comprised from relatively healthy subjects from the general population whereas previous studies had a disease positive NASH cohort.

A relationship between self-reported frequency of beef consumption of liver iron was observed. It is established that there is a significant relationship between red meat consumption and serum ferritin levels [46], and that overfeeding animals with dietary iron causes an increase in liver iron [47]. Furthermore, there is a positive association between red meat intake and liver cancer and chronic liver disease [48, 49]. Although the results presented here are preliminary, the UK Biobank data will allow further investigation of the impact of other dietary factors which have been suggested to have an influence on liver iron and liver health outcomes.

### Limitations

There are several limitations that may impact on the extrapolation of the data obtained from this UK Biobank population to the wider population. It has been shown that the UK Biobank population are somewhat ‘healthier’ that the general UK population, furthermore the age range is limited and there is relatively little ethnic diversity [50].

A further limitation of the current study is the paucity of serum ferritin measurements. Given their general use in assessing iron status in subjects with NAFLD and their potential to add to differentiation between individuals with DIOS and other forms of iron overload [11], they would have been a valuable addition to this population study. However, this marker has not yet been made available by the UKBiobank. In future it is envisaged that blood and other biomarker will be used in combination with the MR measurements described in this paper to aid the development of less complex and less expensive non-invasive tools for liver iron assessment.

MRI measurement of liver iron based on acquisition of T2* or R2*, whilst having the advantage of being fast and non-invasive, can struggle to obtain reliable measurements in very high levels of liver iron content, since the MR signal decays very rapidly. Ideally multi-echo sequences with sufficient echoes, and a short initial echo time need to be employed, but logistic and technical limitations do not make this approach feasible for “large data” studies.

A further confounding factor which may influence the accurate assessment of liver iron relates to the presence of fat infiltration in the liver, since fat-water signal cancellation effects [18] are a particular problem for the type of T2*-Dixon sequence employed in the present study [18, 51, 52]. This issue may be overcome by the use of the IDEAL sequence [53], which addresses these effects by separating out water and fat signals.

Values here are derived from ROIs placed by trained analysts. While more automated approaches are in development, currently human interaction is required to determine appropriate parts of the image to use. However, intra- and inter-rater reliability is excellent for this analysis, as demonstrated by the ICC and Bland-Altman analysis.

### Conclusions

In this paper we show that population studies measuring liver iron are not only feasible, but can now become part of the ever expanding “big data” consortium. As more data is released from the UK Biobank, future studies on liver iron will enable more in-depth investigation on gene-environment interaction, as well as the impact of various lifestyle factors. Moreover, it will provide unique insights to better inform future patient stratification and treatments for elevated liver iron levels. This study has been able to uniquely assess the combined burden of fatty liver and elevated liver iron levels within the UK Biobank population. We have reported that a significant proportion of this population has fatty liver (20.7%) and elevated liver iron levels (4.8%); with 1.3% of the total population having both elevated levels of fat and iron. It has been cautioned in recent years that the UK health system is not prepared for the current liver disease epidemic, with the Lancet Commission for Liver Disease making a series of recommendations in its 2014 report [54]. The numbers of individuals with abnormal levels for two key liver disease risk factors are significant, and potentially represent millions in the wider population. Therefore both liver fat and iron are noteworthy within the liver field’s future research priorities and following public health response.

## Supporting information

S1 Appendix Describes the assessment of repeatability and reproducibility of liver iron measurements included in the manuscript. (DOCX)

S1 Table Describes the published formulas used in previous publications for converting R2* at 1.5T to concentration of liver iron in mg/g. (DOCX)

S2 Table Shows the summary statistics for measurement of liver iron within the UK Biobank cohort expressed T2* (1.5T), R2* (1.5T) and as mg/g. The impact of applying the various formula described in S1 Table on the measurement of liver iron in mg/g are also detailed.
(DOCX)

S3 Table Correlation coefficients for variables. In the univariate models, a model was fitted for each variable, with liver iron as the dependent variable, and only the variable of choice as the independent variable. In the multivariate model, a single model was fitted, with liver iron as the dependent variable, and all variables in the table as independent variables.
(DOCX)

S4 Table Impact of using different R2* cut-offs to indicate iron overload on incidence of the condition within the UK Biobank population. (DOCX)

S1 Fig Describes the distribution of T2* within the UK Biobank population. Median value (24.18 ms) shown in red, mean (23.96 ms) in blue. Values < 15.9ms represent overloaded individuals.
(DOCX)
