# Supplementary material for: Measurement of liver iron by magnetic resonance imaging in the UK Biobank population
Source: PLoS One. 2018 Dec 21;13(12):e0209340. doi: 10.1371/journal.pone.0209340 (PMC6303057; doi:10.1371/journal.pone.0209340)
Supplement: S1 Appendix — (DOCX) [file pone.0209340.s001.docx]

## **S1 Appendix - Repeatability and reproducibility**

Repeatability and reproducibility was assessed with intra-class correlation (ICC) [28] and Bland-Altman analyses [29]. In both cases, iron values were log transformed before analysis, so that differences were homoscedastic (i.e. the variability of the differences was the same across the range of measurements). When an antilog transform is applied to the results from log transform analysis, the calculated Limits of Agreements correspond to a percentage difference in the two values [29]. For instance, limits of agreement of 90% and 110% for a 1.0 mg/g value correspond to 95% confidence that a second analysis of the same data would provide a value between 0.9 and 1.1mg. For a 2mg/g value, those limits would be 1.8mg/g and 2.2mg/g.

Intra- and Inter-rater reliability was calculated for six analysts. Each analyst performed analysis on 56 randomly selected datasets twice on different days. An inter-rater ICC was calculated using the first analysis by each analyst. In addition, Bland-Altman analysis was performed to calculate intra- and interrater Limits of Agreement for each analyst and each pair of analysts.

## Intra- and Inter- analyst repeatability

For the six analysts, ICC analysis of the log transformed iron values gave two results: an ICC (2,1) of 0.98 and an ICC (3,1) of 0.99 [25].

In the intra-rater Bland Altman analyses, the median upper limit of agreement was 107.6% (range 105.6%-112.5%), the median bias was 100.1% (range 99.7%-101.0%), and the median lower limit of agreement was 93.4% (range 89.0%-94.7%). For the inter-rater analyses the median upper limit of agreement was 109.2% (range 106.9%-120.2%), the median bias was 99.7% (range 96.2%-104.1%), and the median lower limit of agreement was 90.3% (range 84.0%-93.5%).
