# Supplementary material for: Measurement of liver iron by magnetic resonance imaging in the UK Biobank population
Source: PLoS One. 2018 Dec 21;13(12):e0209340. doi: 10.1371/journal.pone.0209340 (PMC6303057; doi:10.1371/journal.pone.0209340)
Supplement: S1 Table — (DOCX) [file pone.0209340.s002.docx]

**S1 Table: Published formulas for converting R2* at 1.5T to concentration of liver iron in mg/g.**

| Publication | R2* to liver iron conversion |
| --- | --- |
| Anderson et al 2001 | 0.0146(R2*)-0.27 |
| Hankins et al 2009 | 0.028(R2*)-0.45 |
| Garbowski et al 2014 | 0.032(R2*)-0.14 |
| Wood et al et al 2005 | 0.0254(R2*)+0.202 |
| Paisant et al 2017 | 0.02886(R2*)+0.08 |
| Henninger et al 2015 (GRE sequence) | 0.024(R2*)+0.277 |
| Henninger et al 2015 (ME Dixon sequence) | 0.0312(R2*)+0.294 |
