# Supplementary material for: Measurement of liver iron by magnetic resonance imaging in the UK Biobank population
Source: PLoS One. 2018 Dec 21;13(12):e0209340. doi: 10.1371/journal.pone.0209340 (PMC6303057; doi:10.1371/journal.pone.0209340)
Supplement: S2 Table — The impact of applying the various formula described in S1 Table on the measurement of liver iron in mg/g are also detailed. (DOCX) [file pone.0209340.s003.docx]

**S2 Table: Summary statistics for liver iron expressed T2* (1.5T), R2* (1.5T) and as mg/g.**

|  | **T2*ms** | **R2* S^-1^** | **liver iron mg/g** (Anderson 2001) | **liver iron mg/g** (Hankins 2009) | **liver iron mg/g** (Garbowski 2014) | **liver iron mg/g** (Wood 2005) | **liver iron mg/g** (Paisant 2017) | **liver iron mg/g** (Henninger)  2015 GRE | **liver iron mg/g** (Henninger)  2017 ME |
| --- | --- | --- | --- | --- | --- | --- | --- | --- | --- |
| **Mean** | 23.96 | 43.84 | 0.37 | 0.78 | 1.26 | **1.32** | 1.35 | 1.37 | 1.66 |
| **St. dev** | 4.60 | 12.53 | 0.18 | 0.35 | 0.40 | **0.32** | 0.36 | 0.31 | 0.39 |
| **5^th^ Percentile** | 16.09 | 32.30 | 0.20 | 0.45 | 0.89 | **1.02** | 1.01 | 1.08 | 1.30 |
| **25^th^ Percentile** | 21.11 | 36.87 | 0.27 | 0.58 | 1.04 | **1.14** | 1.14 | 1.20 | 1.44 |
| **Median** | 24.18 | 41.35 | 0.33 | 0.71 | 1.18 | **1.25** | 1.27 | 1.31 | 1.58 |
| **75^th^ Percentile** | 27.12 | 47.36 | 0.42 | 0.88 | 1.38 | **1.40** | 1.45 | 1.46 | 1.77 |
| **95^th^ percentile** | 30.96 | 62.17 | 0.64 | 1.29 | 1.85 | **1.78** | 1.87 | 1.83 | 2.23 |
