# Supplementary material for: Measurement of liver iron by magnetic resonance imaging in the UK Biobank population
Source: PLoS One. 2018 Dec 21;13(12):e0209340. doi: 10.1371/journal.pone.0209340 (PMC6303057; doi:10.1371/journal.pone.0209340)
Supplement: S3 Table — In the univariate models, a model was fitted for each variable, with liver iron as the dependent variable, and only the variable of choice as the independent variable. In the multivariate model, a single model was fitted, with liver iron as the dependent variable, and all variables in the table as independent variables. (DOCX) [file pone.0209340.s004.docx]

**S3 Table: Correlation coefficients for variables.**

| Variable | Univariate models | | Multivariate model | |
| --- | --- | --- | --- | --- |
|  | Effect estimate | P value | Effect estimate | P value |
| Age | 0.0051 | <2x10^-16^ | 0.0042 | <2x10^-16^ |
| Sex – Female | 0 | (Reference) | 0 | (Reference) |
| Sex - Male | 0.0586 | <2x10^-16^ | 0.0342 | 5.09x10^-7^ |
| Log(liver fat) | 0.0601 | <2x10^-16^ | 0.0639 | <2x10^-16^ |
| BMI | 0.0033 | 1.9x10^-5^ | -0.0046 | 6.3x10^-7^ |
| Beef intake - Never | 0 | (reference) | 0 | (reference) |
| Beef intake – less than once a week | 0.0799 | 4.0x10^-13^ | 0.062 | 1.1x10^-8^ |
| Beef intake – once a week | 0.1082 | <2x10^-16^ | 0.086 | 1.2x10^-13^ |
| Beef intake – more than once a week | 0.1218 | 5.3x10^-16^ | 0.102 | 8.5x10^-12^ |
| Intercept | NA | NA | 1.0361 | <2x10^-16^ |
