# Supplementary material for: Measurement of liver iron by magnetic resonance imaging in the UK Biobank population
Source: PLoS One. 2018 Dec 21;13(12):e0209340. doi: 10.1371/journal.pone.0209340 (PMC6303057; doi:10.1371/journal.pone.0209340)
Supplement: S4 Table — (DOCX) [file pone.0209340.s005.docx]

**S4 Table: Impact of using different R2* cut-offs to indicate iron overload on incidence of the condition within the UK Biobank population.**

| Publication | Reported R2* cut-off for iron overload (sec^-1^) | Incidence of iron overload within the UK Biobank |
| --- | --- | --- |
| Kuhn et al [33] | >41 | 51.5% |
| Henninger et al [12] | 57 | 7.5% |
| Paisant et al [22] | 65 | 4.2% |
| Henninger et al [15] | <70 | 3.0% |
