# Supplementary material for: Measurement of liver iron by magnetic resonance imaging in the UK Biobank population
Source: PLoS One. 2018 Dec 21;13(12):e0209340. doi: 10.1371/journal.pone.0209340 (PMC6303057; doi:10.1371/journal.pone.0209340)
Supplement: S1 Fig — Median value (24.18 ms) shown in red, mean (23.96 ms) in blue. Values < 15.9ms represent overloaded individuals. (DOCX) [file pone.0209340.s006.docx]

**S1 Fig: Distribution of T2* within the UK Biobank population.**

**
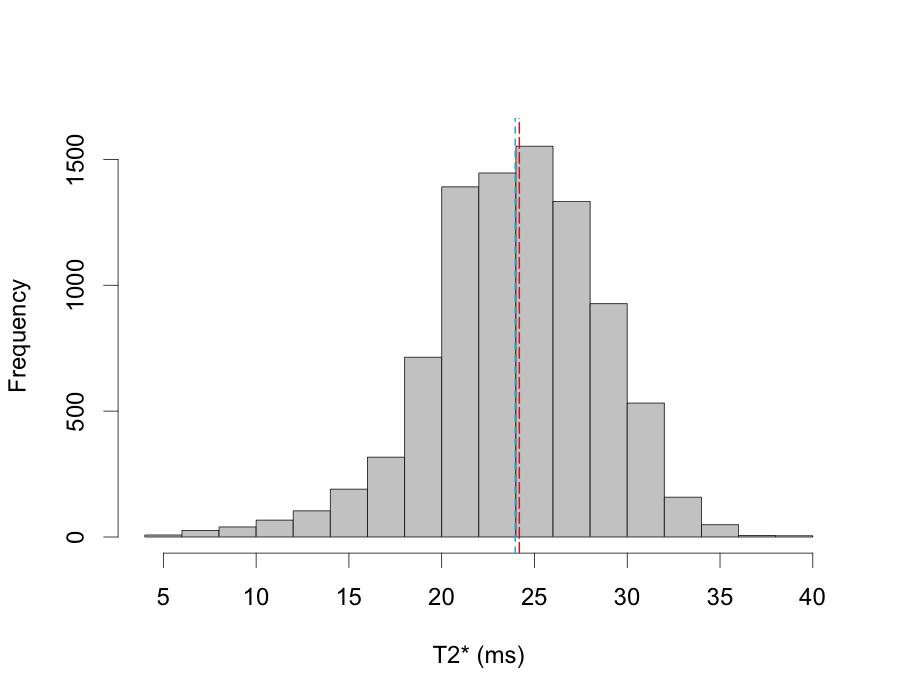
**

Median value (24.18 ms) shown in red, mean (23.96 ms) in blue. Values < 15.9ms represent overloaded individuals.
